# Primary cranial mediastinal hemangiosarcoma in a young dog

**DOI:** 10.1186/2046-0481-67-15

**Published:** 2014-07-27

**Authors:** Hun-Young Yoon, Hye-Mi Kang, Mi-Young Lee

**Affiliations:** 1 Department of Veterinary Surgery, College of Veterinary Medicine, Konkuk University, Seoul 143-701, South Korea; 2 Busan Animal Medical Center, Busan 611-800, South Korea; 3 Department of Veterinary Radiology and Diagnostic Imaging, College of Veterinary Medicine, Konkuk University, Seoul 143-701, South Korea

**Keywords:** Primary cranial mediastinal hemangiosarcoma, Young dog, Blunt dissection, Debridement

## Abstract

Primary cranial mediastinal hemangiosarcomas are uncommon tumors. A 30-kg, 2-year-old, intact female German shepherd was presented for evaluation of cachexia and respiratory distress of a few days’ duration. Lateral radiographic projection of the thorax revealed significant pleural effusion. Computed tomography revealed a cranial mediastinal mass effect adjacent to the heart. On surgical exploration, a pedunculated mass attached to the esophagus, trachea, brachiocephalic trunk, left subclavian artery and cranial vena cava without attachment to the right atrium and auricular appendage was removed and debrided by use of blunt dissection and dry gauzes, respectively. Histopathology results described the cranial mediastinal mass as hemangiosarcoma. At 8 months and 5 days post-operatively, the patient died. Primary cranial mediastinal hemangiosarcomas, although a seemingly rare cause of thoracic pathology in young dogs, should be considered in the differential diagnosis for pleural effusion and soft tissue mass effect in the cranial mediastinum. This is the first case report in a dog to describe primary cranial mediastinal hemangiosarcoma.

## Background

Hemangiosarcoma (HSA) is a highly malignant tumor that originates from vascular endothelium [1]. The most common primary sites of HSA include the spleen (28% to 50 V), right atrium/auricular appendage (3% to 50%) and skin or subcutaneous tissue (13%) [2-4]. Other primary sites of HSA include pericardium, liver, muscle, lung, bone, kidney, central nervous system, peritoneum, oral cavity, eye, prostate, uterine remnant, penis and other non-parenchymal sites [5-13]. The mean age range of occurrence in dogs is 8 to 13 years, and German shepherds, golden retrievers and Labrador retrievers are overrepresented [13-15]. Echocardiography and computed tomography (CT) are considered to be good diagnostic tools; however, histological evaluation is necessary for a definitive diagnosis [3]. Treatment usually includes partial pericardiectomy or tumor resection for cardiac HSA [3]. To the authors’ knowledge, this is the first case report in a dog to describe primary cranial mediastinal HSA. The purpose of this case report is to describe the clinical presentation, diagnostic approach and palliative surgical management of primary cranial mediastinal HSA in a young dog.

## Case presentation

**Figure 1:**
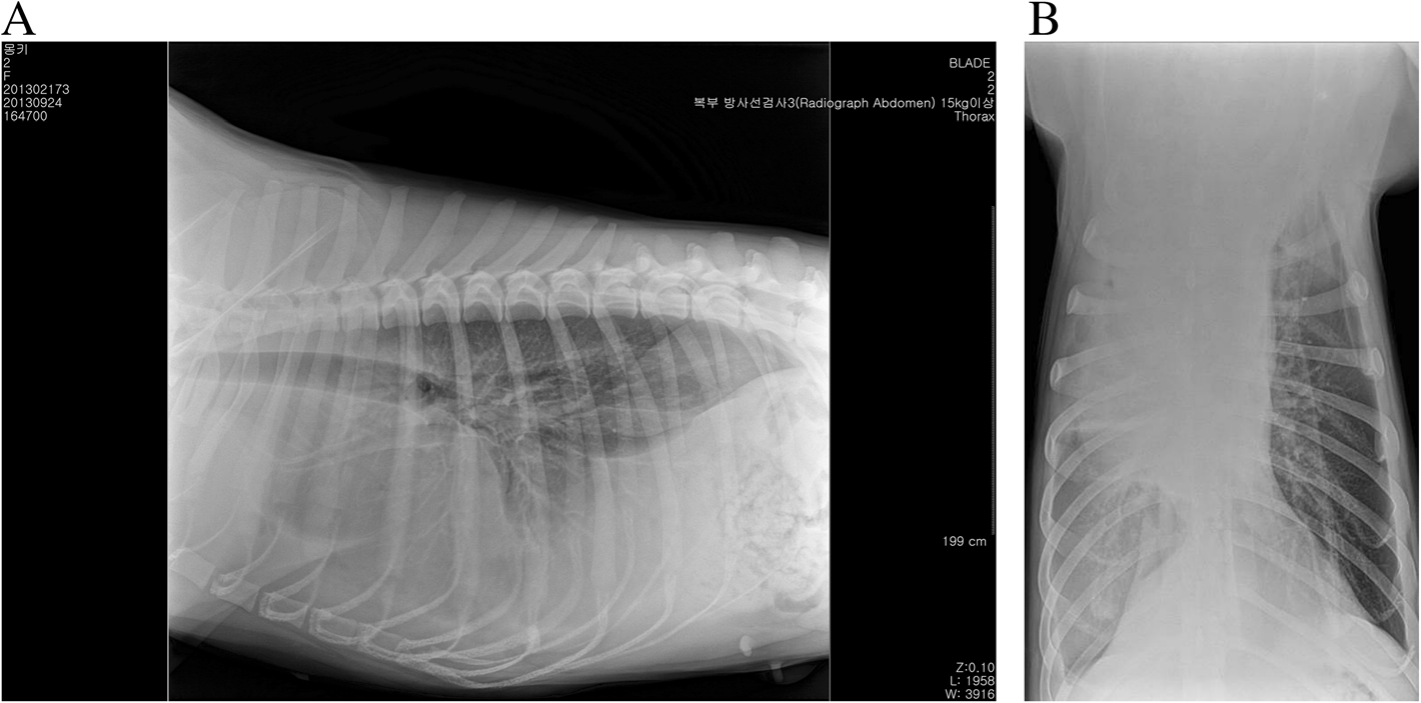
**Preoperative radiographic view. (A)** Lateral projection of the thorax reveals significant accumulation of pleural effusion. **(B)** Ventrodorsal projection of the thorax reveals a widened cranial mediastinum with increased soft-tissue density.

**Figure 2:**
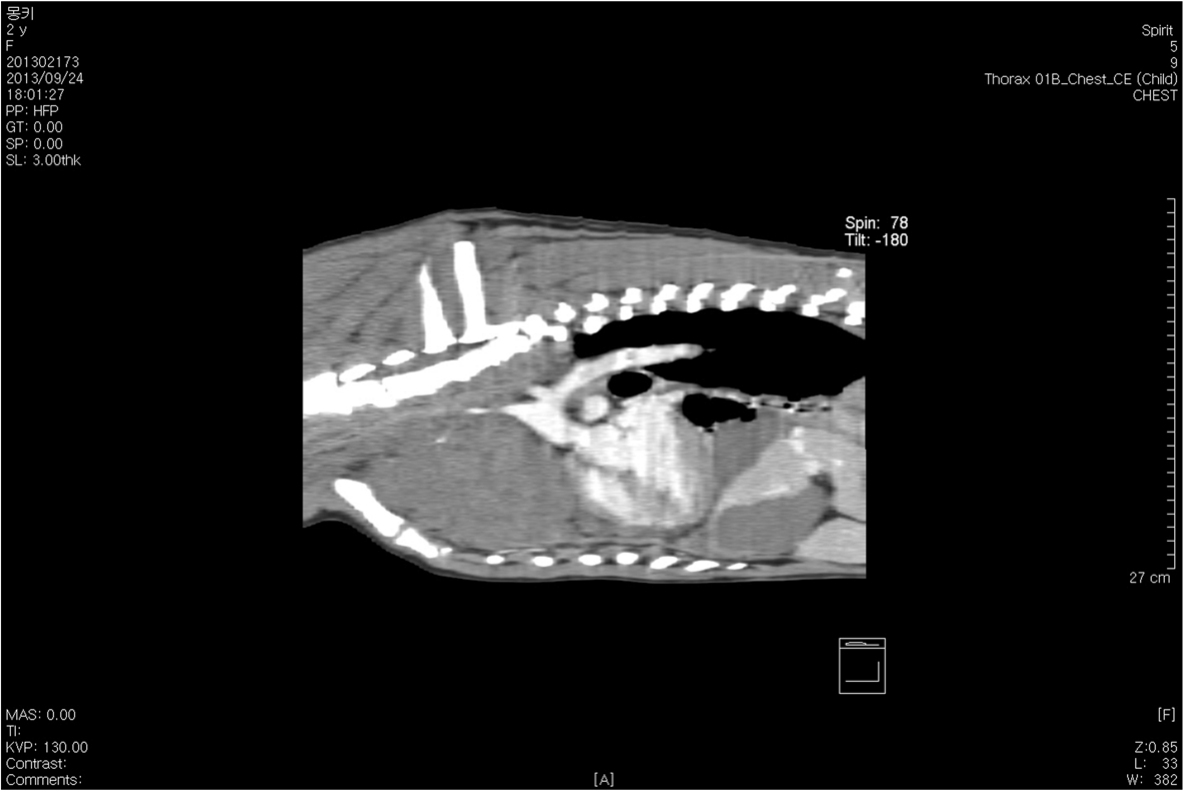
**Preoperative sagittal computed tomography post-contrast image.** A mass effect is present in the cranial mediastinum, adjacent to the heart. The mass is approximately 9 cm in height, 8 cm in width and 12 cm in length.

**Figure 3:**
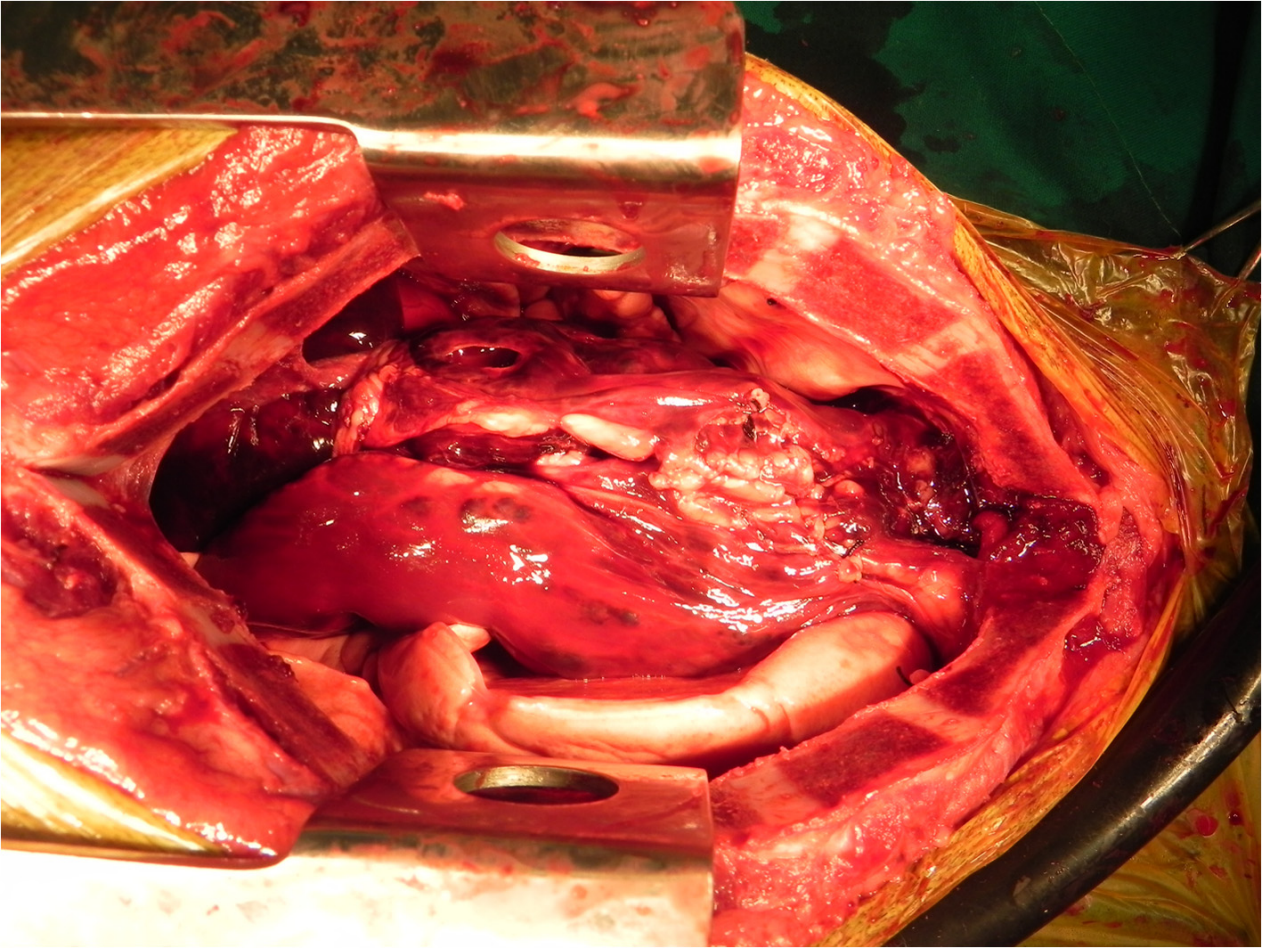
**Intraoperative photograph.** A pedunculated mass located in the cranial mediastinum is attached to the surrounding organs including the esophagus, trachea, brachiocephalic trunk, left subclavian artery and cranial vena cava.

**Figure 4:**
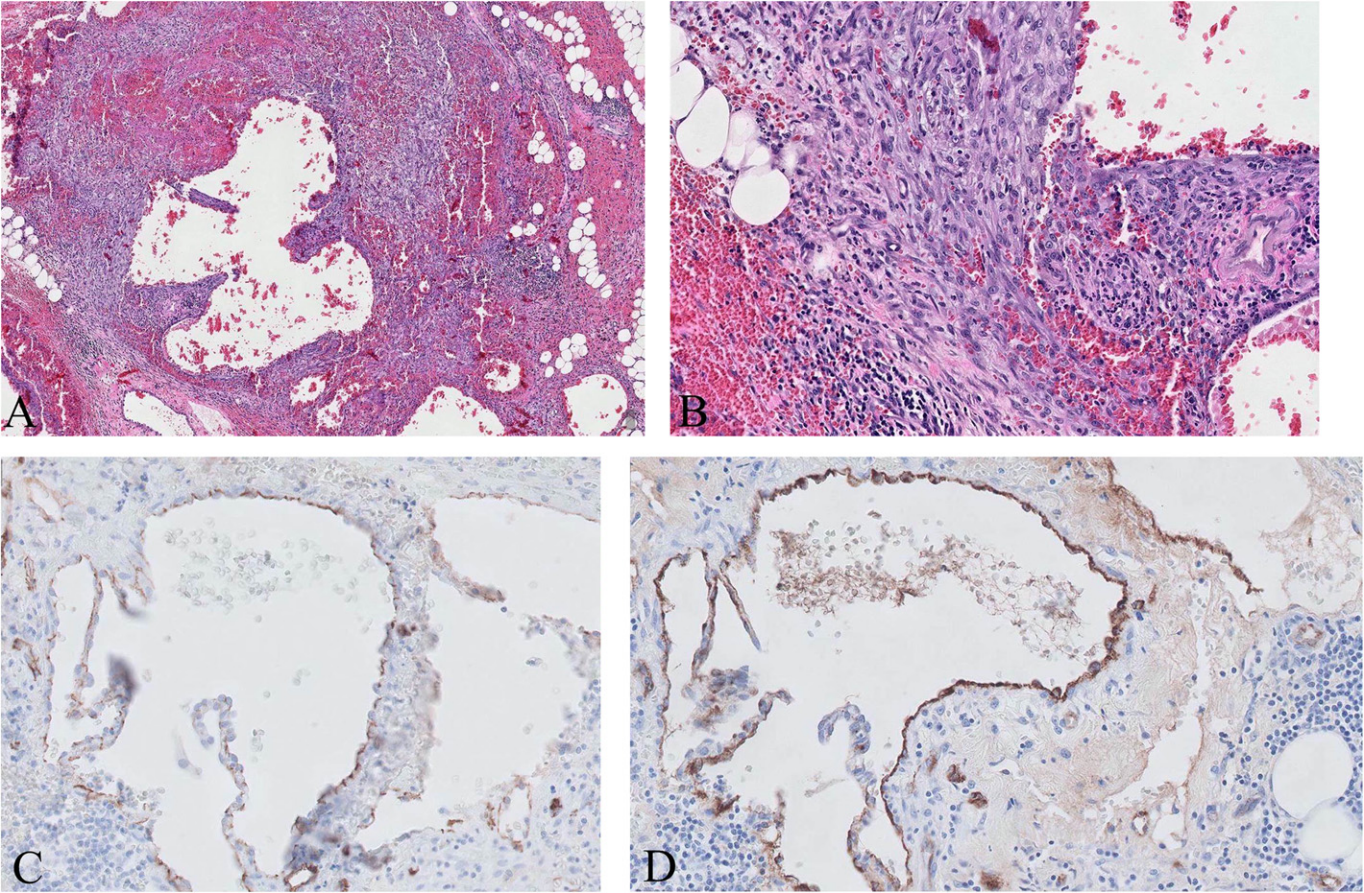
**Cranial mediastinal hemangiosarcoma. (A)** The sample consists of residual fat with some fibrous stroma and there are multifocal, extensive areas of hemorrhage. Surrounding areas of hemorrhage are composed of nests of neoplastic endothelial cells, 50X H&E stain objective. **(B)** The cells are tightly packed with minimal amphophilic cytoplasm and hyperchromatic plump oblong nuclei with coarsely stippled chromatin and a single nucleolus, 200X H&E stain objective. **(C)** CD31 immunohistochemistry shows immunoreactivity of cells lining vascular channels. Atypical spindle cells are positive for CD31, 20X CD31 stain objective. **(D)** Factor VIII immunohistochemistry shows immunoreactivity of cells lining vascular channels. Atypical spindle cells are positive for factor VIII, 20X factor VIII stain objective.

**Figure 5:**
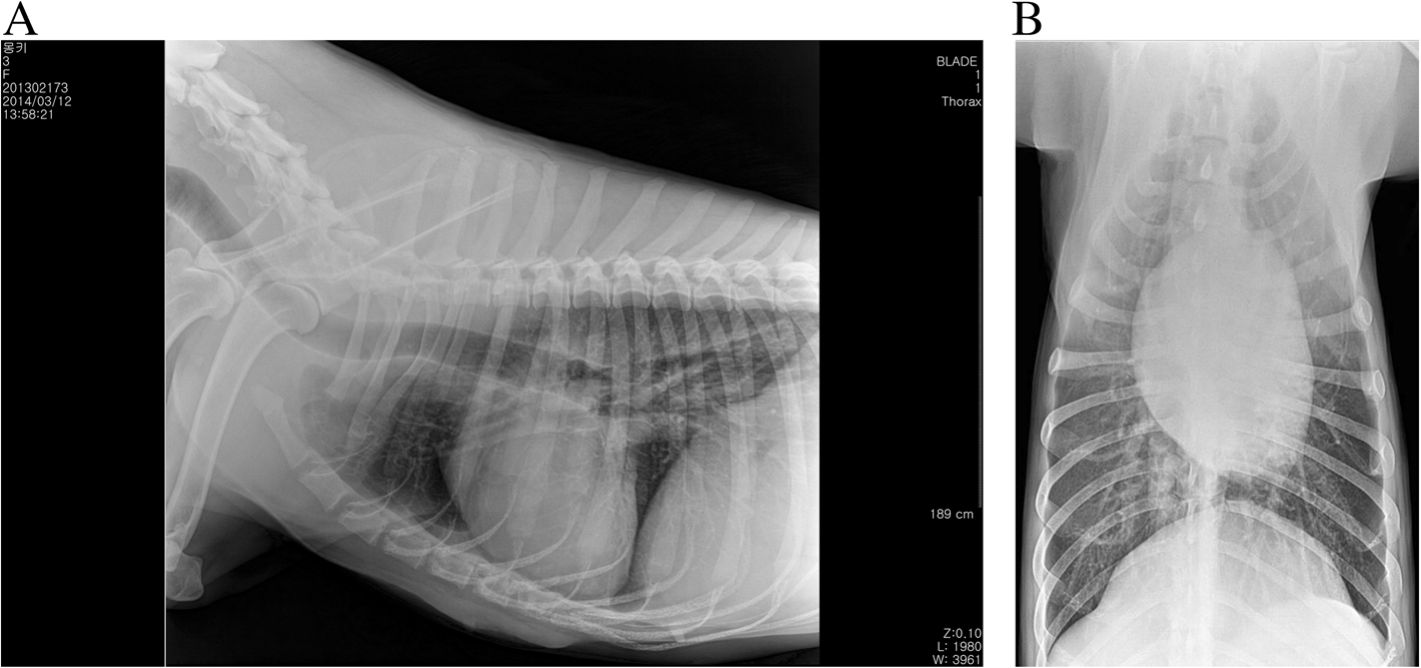
**Postoperative radiographic view at 6 months.** Lateral **(A)** and ventrodorsal **(B)** projections of the thorax reveal no pleural effusion and no evidence of increased soft-tissue density associated with widening of the cranial mediastinum.

A 30-kg, 2-year-old, intact female German shepherd was presented for evaluation of cachexia and respiratory distress of a few days’ duration. On physical examination, the dog showed labored breathing with a respiratory rate of approximately 90/minute. A low hematocrit (28.9%) was identified on complete blood count profiling. At 189 × 10^3^/μL, platelets were in the low-normal range (reference range: 180 to 500 × 10^3^/μL). A urinalysis and serum biochemistry and coagulation profiles were unremarkable. Lateral and ventrodorsal radiographic projections of the thorax revealed significant accumulation of pleural effusion and widening of the cranial mediastinum with increased soft-tissue density (Figure 1A and B). Sanguineous fluid (700 ml) was removed under ultrasound-guided thoracocentesis. Pleural effusion and ultrasound-guided fine needle aspiration cytology of the mass predominantly revealed erythrocytes and no tumor cells. On auscultation, there was no evidence of muffled heart sound. A thoracic ultrasound and CT scan revealed a mass effect in the cranial mediastinum adjacent to the heart (Figure 2). The mass was approximately 9 cm in height, 8 cm in width and 12 cm in length. There was no evidence of invasion into the cranial vena cava, brachiocephalic trunk or left subclavian artery. An abdominal CT scan was performed to rule out other differentials and demonstrated no evidence of masses on the spleen, liver and other sites. The CT scan diagnosis was cranial mediastinal soft tissue neoplasm.Surgical exploration of the thorax was performed through median sternotomy. The upper half of the manubrium and the xiphoid process were left intact. A pedunculated mass located in the cranial mediastinum was attached to the surrounding organs including the esophagus, trachea, brachiocephalic trunk, left subclavian artery and cranial vena cava without attachment to the right atrium, right auricular appendage, pericardium and heart base (Figure 3). The mass was removed by blunt dissection from the organs to which it was attached, including the esophagus, trachea, brachiocephalic trunk, left subclavian artery and cranial vena cava. The internal thoracic artery and vein were sacrificed for aggressive surgical removal of the mass. Segments of the residual mass attached to the organs were debrided with dry gauze. The removed mass was submitted for microscopic evaluation. The thoracic cavity was then lavaged with warmed sterile saline. A left-sided thoracostomy tube was placed for drainage of anticipated post-operative pleural effusion. Sternotomy closure was accomplished with a cruciate suture pattern using 1 polydioxanone (PDS II; Ethicon, Inc., Somerville, NJ, USA).Formalin-fixed, paraffin-embedded tissue sections were stained with hematoxyline and eosin. The sample was blindly submitted to two different pathologists. Pathologist 1 reported that the sample consisted of residual fat with some fibrous stroma. There were multifocal, extensive areas of hemorrhage. These were associated with coagulation necrosis. Surrounding areas of hemorrhage were composed of nests of neoplastic endothelial cells. The cells were tightly packed with minimal amphophilic cytoplasm and hyperchromatic plump oblong nuclei with coarsely stippled chromatin and a single nucleolus (Figure 4A and B). Pathologist 2 reported that the sample consisted of collagenous connective tissue in which there was a poorly demarcated, unencapsulated neoplasm composed of vascular channels separated by abundant hemorrhage and fibrin. The neoplastic cells were spindle to plump in shape with abundant amphophilic cytoplasm and indistinct cell borders. The nuclei were oval with reticular chromatin and occasionally contained a single prominent nucleolus. There were multifocal areas of necrosis. Histopathology results described the cranial mediastinal mass as HSA. For immunohistochemistry, CD31 and factor VIII were used as HSA markers. Atypical spindle cells were positive for CD31 and factor VIII, which supported HSA, as described above (Figure 4C and D).The thoracostomy tube was removed 8 days post-operatively when the amount of serosanguineous fluid production was 1.7 ml/kg/day. The patient’s hematocrit level was 34.3% on complete blood count profiling, 7 days post-operatively. The owner did not consent to chemotherapy. Recheck examinations were scheduled monthly. At 6 months, lateral and ventrodorsal radiographic projections of the thorax revealed no pleural effusion and no evidence of increased soft-tissue density associated with widening of the cranial mediastinum (Figure 5A and B). However, it was not possible to determine whether there was macroscopic metastasis. The owner did not consent to further CT scan to identify macroscopic metastasis. There was no evidence of cachexia and respiratory distress, 6 months post-operatively. At 8 months and 5 days post-operatively, the patient died with respiratory distress of a week’s duration. The owner declined a postmortem examination.

## Conclusions

The right atrium and auricle, pericardium, heart base and left-sided cardiac chambers are primary cardiac sites for HSA affecting the thoracic cavity in dogs, even though primary HSA can develop in any vascularized site in the thoracic cavity [3,16]. Primary cardiac HSAs are dominantly located in the right atrium and right auricular appendage in dogs [3,16]. In humans, the right atrium is the most common site for primary cardiac angiosarcoma, a vascular neoplasm synonymous with HAS, and other primary sites for HSA are rare in the thoracic cavity [17]. Clinical signs of right atrial or right auricular HSA are usually related to pericardial effusion, cardiac tamponade and signs of right-sided heart failure [18]. In the case reported here, no muffled heart sounds and abdominal fluid wave were identified on auscultation after ultrasound-guided thoracocentesis and physical examination, respectively. On CT evaluation and gross examination, the cranial mediastinal mass did not appear to originate from the right atrium or right auricle. Instead, the mass appeared to originate from another primary site within the cranial mediastinum. In cats, HSAs are rare, accounting for less than 2% of nonhematopoietic tumor, and cardiac HSA is extremely uncommon [19]. Interestingly, in a retrospective study of feline HSAs, one intrathoracic tumor out of 10 visceral HSAs was mediastinal HSA [19].

Intrathoracic tumors rarely occur in the cranial mediastinum in dogs [20]. Differential diagnoses for cranial mediastinal masses include thymoma, lymphosarcoma, ectopic thyroid or parathyroid tissue, chemodectoma, metastic neoplasia, granuloma and thymic branchial cyst [20]. In the case reported here, because an abdominal CT demonstrated no evidence of masses on the spleen, liver and other sites, thymoma and lymphosarcoma were the primary differential diagnoses for a cranial mediastinal mass. However, histopathology results described the cranial mediastinal mass as hemangiosarcoma. Histopathologic differentiation of HSA from other intrathoracic tumors is essential for an accurate diagnosis. Given our current results, primary cranial mediastinal HSA should be considered in the differential diagnosis for a mass in the cranial mediastinum.

For palliative management of cardiac HSA, subtotal pericardiectomy can be an option to alleviate cardiac tamponade if surgical excision of the cardiac HSA is either not feasible or recommended due to presence of metastases [3]. In the case reported here, the mass, which was attached to the esophagus, trachea, brachiocephalic trunk, left subclavian artery and cranial vena cava, was removed by use of blunt dissection. Dry gauzes were used to debride the residual mass attached to the organs. Tumor removal with blunt dissection and dry gauzes could be an option for palliative management of mediastinal HSA.

Canine HSAs occur predominantly in older dogs and rarely in younger dogs [18,21,22]. Previous reports have described the mean age range at the time of diagnosis in dogs as 3 to 15 years, 8 to 13 years or 9 to 12 years [3,18,23]. In the case reported here, the age at the time of diagnosis was 2 years. A previous report has described a young patient age to be associated with prolonged survival [24].

Primary cranial mediastinal hemangiosarcomas, although a seemingly rare cause of thorax pathology in young dogs, should be considered in the differential diagnosis for pleural effusion and soft tissue mass effect in the cranial mediastinum.

## Competing interests

The authors declare that they have no competing interests.

## Authors’ contributions

HY contributed to the case by means of case management including surgical performance and drafted the manuscript. HM participated in case management. MY performed the imaging evaluation of this case by means of radiology and CT. All authors read and approved the final manuscript.
